# Computational Fluid Dynamics (CFD) in Arteriovenous (AV) Graft Implantation Through End-to-Side Anastomosis with Varying Tube Diameters Across Different Vascular Access Locations for Dialysis Treatment

**DOI:** 10.3390/medicina60101704

**Published:** 2024-10-17

**Authors:** Roland Jayson Panganiban, Reniela Redem Lictaoa, Martin Lance Mesia, Jordan Angelo Amorado, Heherson Cabrera

**Affiliations:** 1 School of Chemical, Biological, and Materials Engineering and Sciences, Mapua University, Manila 1002, Philippines; rjmpanganiban@mymail.mapua.edu.ph (R.J.P.); rrclictaoa@mymail.mapua.edu.ph (R.R.L.); mlvmesia@mymail.mapua.edu.ph (M.L.M.); jadamorado@mymail.mapua.edu.ph (J.A.A.); 2 School of Health Sciences, Mapua University, Brgy. Sta. Cruz, Makati 1205, Philippines

**Keywords:** computational fluid dynamics, arteriovenous graft implantation, end-to-side anastomosis

## Abstract

*Background/Objectives:* Arteriovenous (AV) graft is a procedure for hemodialysis performed in the arm. Optimizing AV graft design is vital to enhance haemodialytic efficiency in patients with kidney disease. Despite being a standard procedure, making it work optimally is still difficult due to various graft diameters and anastomosis configurations, which have limited studies. This research aims to find the ideal AV graft tube diameter on blood flow and pressure gradients and the ideal body site for AV graft implantation and to study their angles for dialysate flow. *Methods:* Nine models were designed in Autodesk Fusion 360 with 40°, 50°, and 60° angles each having 2 mm, 5.1 mm, and 14.5 mm diameters, all following specific equations on continuity, momentum (Navier-Stokes Equation)), and the Reynolds Stress Model (RSM). The CFD simulation of these models was performed in ANSYS Fluent with an established parameter of 0.3 m/s inlet velocity and stiff/no-slip graft and artery wall boundary condition. *Results:* As a result, the design with a diameter of 14.5 mm and a 40° angle was overall the most ideal in terms of minimal wall shear stress and turbulence. *Conclusions:* Thus the brachiocephalic area or the forearm is calculated to be the most optimal implantation site. Additionally, varying angles do affect dialysate flow, as smaller values cause less stress.

## 1. Introduction

In the realm of nephrology, arteriovenous fistula (AV graft) design, typically located in the arm at the radiocephalic, brachiocephalic, or brachiobasillic veins, is a fundamental procedure for hemodialysis [1]. This surgically crafted connection between an artery and vein serves as a permanent lifeline for patients with advanced kidney disease. Hemodialysis, a blood filtration treatment, is essential for these patients as their kidneys are no longer able to adequately remove waste products and excess fluids from the body [1]. An ideal AV graft configuration minimizes resistance to blood flow, maximizing the clearance of these waste products by the dialysate fluid used in hemodialysis. However, this remains a challenge [2].

The researchers create an AV graft that not only ensures efficient blood flow but also minimizes the risk of complications like stenosis (narrowing) and thrombosis (blood clot formation) within the graft itself [3,4,5]. This research utilizes a modern technique to enhance AV graft functionality, hence facilitating improvements in patient outcomes. The researchers are interested in studying the influence of varying diameters along the AV graft on the flow dynamics and pressure gradients within the graft. By understanding how these changes affect blood flow, the researchers aim to address the significant challenges associated with AV graft function. Specifically, the researchers are investigating how AV graft tube diameter modifications across different locations, including the radio cephalic, brachiocephalic, and brachioradialis veins, influence essential flow parameters such as vascular resistance and wall shear stress. Wall shear stress refers to the frictional force exerted by the flowing blood on the walls of the graft. Understanding how these parameters change with varying diameters is essential for optimizing AV graft design. Additionally, the study also seeks to evaluate the impact of varying tube diameters on the efficiency of dialysate flow across the end-to-side anastomosis, the critical junction where the artery and vein connect. Dialysate fluid is a cleansing solution for hemodialysis to remove waste products from the blood. Good dialysate fluid flow across the anastomosis is vital for good waste removal.

**Figure 1:**
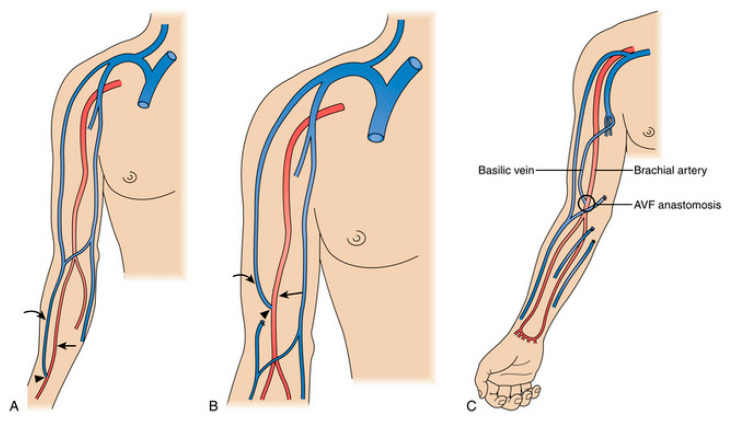
Hemodialysis access locations. (**A**) Endogenous radio cephalic arteriovenous graft. (**B**) Endogenous brachiocephalic graft. (**C**) Endogenous transposed brachiobasilic graft [6].

Upon establishing the objectives of this study, the researchers focused on a critical aspect of AV graft design: the location of the anastomosis and the point of connection between the artery and vein. As illustrated in Figure 1, AV graft creation typically involves surgically connecting an artery to a vein at one of these three common locations: the radio-cephalic (Figure 1A), brachiocephalic (Figure 1B), or brachioradialis (Figure 1C). This anastomosis is a critical element for efficient blood flow within the fistula. The arrow represents the inflow radial/brachial artery, the arrowhead represents the arteriovenous anastomosis, and the outflow represents the outflow cephalic/transposed basilic vein. Discovering and understanding the varying graft diameters at these anatomical sites are essential for optimizing AV graft design.

Current research suggests specific diameter ranges for optimal AV graft function. In the radio cephalic AV graft, the ideal radial artery diameter for maximum performance is at least 2 mm [7]. Similarly, the cephalic vein diameter should be at least 2 mm (without augmentation) for the best outcomes [7]. A cephalic vein diameter below 1.5 mm is generally not recommended [7]. These considerations are crucial for informing decisions about AV graft placement and design. For the brachiocephalic vein, research suggests an average diameter of around 14.5 to 17.0 mm [8]. This information is valuable for guiding decisions about AV graft placement in this location. On the contrary, limited data exists on the optimal diameter specifically for the basilic vein used in brachioradialis AV graft creation. However, one study suggests that the basilic vein may be amenable to enlargement using percutaneous transluminal angioplasty (PTA) with a drug-eluting balloon (DEB) [4]. This minimally invasive procedure successfully dilated the basilic vein outflow diameter by up to 41% compared to a control group, with a pre-intervention diameter of around 3.3 mm. This enlargement potentially improved AV graft patency rates [9]. While the ideal size for an unfused basilic vein is not definitively established, this study suggests that veins with a diameter around 3.3 mm may be suitable candidates for PTA with DEB to achieve acceptable diameters for AV graft creation, likely in the range of approximately 4.6 to 4.7 mm [9].

By demonstrating the importance of vessel diameter in AV graft creation, the researchers explore the three main objectives of this study. First, the researchers will analyze how variations in AV graft diameter impact blood flow and pressure gradients at different access points within the vascular system. This analysis will provide valuable insights into how varying diameters affect flow characteristics throughout the AV graft. Second, to identify the optimal location within the body for AV graft implantation based on the complex interplay between flow dynamics and wall shear stress induced by varying tube diameters. Identifying optimal placement sites has the potential to significantly improve AV graft function and treatment outcomes for patients. Finally, to evaluate the influence of the end-to-side anastomosis graft design, particularly the angle of configuration, on the efficiency of dialysate flow from the artery to the vein. The angle of configuration at the anastomosis may impact flow patterns and the efficiency of waste removal.

The findings of this study hold significant knowledge for revolutionizing AV graft design and placement strategies. By exploring the important relations between AV graft diameter, flow dynamics, and graft design in a more in-depth method, the researchers gain a deeper understanding of the connection between these factors and what constitutes an optimal AV graft. This newfound knowledge has the potential to revolutionize how AV grafts are created, leading to more efficient and durable access points for patients with advanced kidney disease who rely on hemodialysis for survival. By optimizing these factors, future AV grafts could experience fewer complications like stenosis and blood clots, improving patient outcomes and quality of life [2].

## 2. Materials and Methods

The implantation of AV grafts associated with three places with various diameters and angles requires computational simulation tools, governing equations, and geometric models in its process and design. It involves the precise determination of fluid characteristics and the establishment of boundary conditions to simulate the dynamics of blood flow within the graft.

### 2.1. Geometric Models

**Figure 2:**
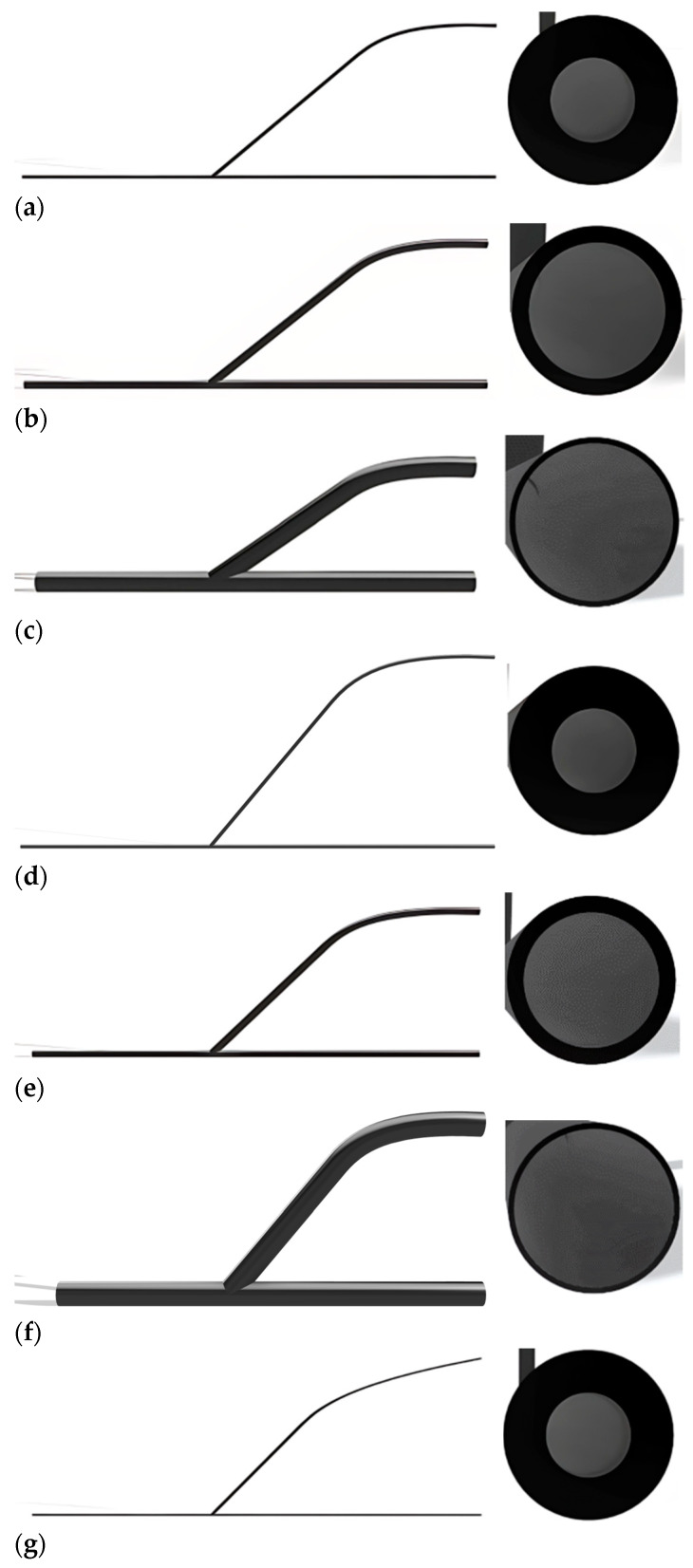

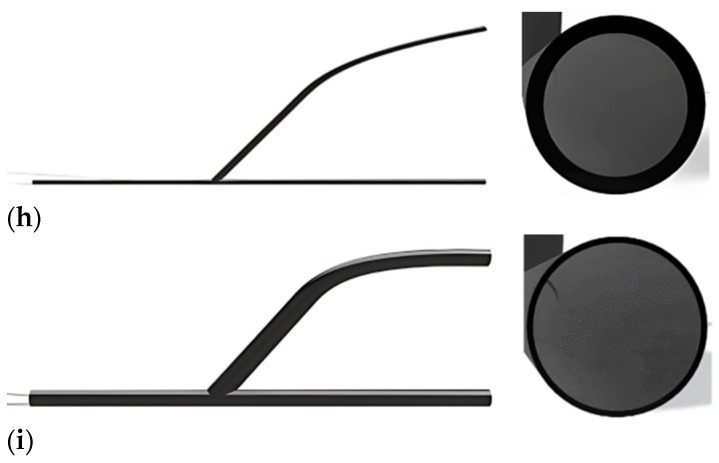
Geometric front view and diameter of the nine anastomosis models. The diameters of the nine models: 2 mm for (**a**,**d**,**g**); 5.1 mm for (**b**,**e**,**h**); and 14.5 mm for (**c**,**f**,**i**). Three angles that show in the geometric front views: 40° for (**a**–**c**), 50° for (**d**–**f**), and 60° for (**g**–**i**).

The AV graft’s geometrical representations were generated using Autodesk Fusion 360. This experiment used an end-to-side (ETS) graft anastomosis with varied tube diameters across many sites. The sizes are determined based on three specific locations: the radio cephalic, brachioradialis, and brachiocephalic veins. The graft and host artery diameters were fixed at 2 mm, 5.1 mm, and 14.5 mm, respectively. The graft length remained constant at 250 mm for all anastomosis angles depicted in Figure 2. Each version was designed with a consistent inner diameter section thickness of 0.5 mm. A sectional analysis was conducted to expose the internal details of the design and eliminate any portions that may block blood flow between the heel and toe along the vein.

The AV graft’s angle of attachment to the artery was varied to investigate the impact of the dialysate flow from the artery to the vein. The measured angles of the simulated blood flow in the artery were 40°, 50°, and 60°, relative to the direction of blood flow. Based on the literature, this hemodynamic information was obtained and analyzed to guarantee that the flow conditions into and out of the graft and the anastomosis junction were properly developed [10]. Each diameter was examined for each anastomosis angle across nine models.

### 2.2. Governing Equations

The governing equations play a crucial role in the study of blood flow using computational fluid dynamics. Governing equations serve as the fundamental basis for CFD analysis and are essential for comprehending, modeling, and evaluating the behavior of blood flow in different physiological and pathological states [11]. The equations that regulate the system are:

Continuity equation:

(1) ∇·ν=0

Navier–Stoke equation (momentum equation):

(2) $$\rho \frac{\partial \nu }{\partial t}+\rho \left(\nu \cdot \nabla \right)\nu =\left(-\nabla p+\mu \nabla^{2}v\right)$$

where *µ* is the velocity vector, *t* is the time, *p* is the pressure, and *ρ* is blood density. The relationship between blood flow and the Navier–Stokes equations is established using the principles of fluid dynamics. This study employed the Navier–Stokes equation to explain the transfer of momentum in the blood, enabling the quantitative modeling of fluid flow. Boundary conditions are prescribed in computational simulations using the Navier–Stokes equations to replicate physiological circumstances in the real world [12]. The process involves specifying the inflow circumstances at the entrance sites of the arteries, the outflow conditions at the exit points of the veins, and determining the characteristics of the vessel walls, like the requirement for no-slip conditions for viscous walls [13].

Reynolds stress model (RSM):

(3) $$\frac{Du_{i}}{Dt}=-\frac{1}{\rho }\frac{\partial \rho }{\partial x_{i}}+v\frac{\partial^{2}u_{i}}{\partial x_{k}^{2}}-\frac{\partial u_{i}^{\prime }u_{k}^{\prime }}{\partial x_{k}}$$

The researchers employ this equation to demonstrate the impact of turbulence intensity on the blood flow within the veins in the given configurations. Blood may transition from a laminar to a turbulent flow. The Reynolds stress model (RSM) is a commonly used turbulence model, especially in the context of using the Navier–Stokes equation. It is essential for studying and interpreting the patterns of blood flow and has an impact on hemodynamic parameters in CFD simulations [14]. Increased RSM can result in higher shear stresses on the walls of vessels, changes in flow patterns, and the occurrence of localized turbulence-related phenomena such as flow separation or recirculation zones [15]. CFD analysis has the potential to enhance efficiency, provide solutions for turbulent flow modeling, and reduce development expenses.

### 2.3. Computation Fluid Dynamics (CFD) Analysis

**Figure 3:**
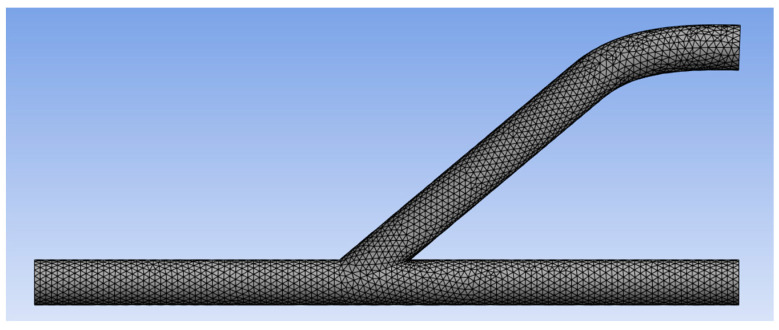
Mesh Model.

A CFD analysis was conducted to determine the most suitable diameter and anastomosis angle, observed wall shear stress (WSS) and turbulence intensity alterations, and appropriate placement for AV graft implantation. Initially, a mesh was generated using the ANSYS fluid flow (fluent) meshing program. All nine AV graft models were composed of fine and tetrahedral core components in the mesh. The objective of this meshing strategy was to provide a refined mesh that accurately captures the physics of the model, ensuring precision, numerical stability, and efficient computing time for CFD simulations [16]. Additionally, mesh boundaries were established for the model to define the inlet (velocity), outlet (pressure), and wall conditions. Figure 3 shows a mesh of the model for a 14.5 mm at 40° graft. A triangular mesh is utilized in the model, which is a program-controlled option in the software.

The fluid flow (fluent) approach in the ANSYS 2023 R2 Teaching Version 23.2 software tool was employed to solve the governing equations of fluid dynamics. The solution set-up was established within specific parameters and conditions. The viscous model was employed with Reynolds stress to quantify the turbulence intensity of the blood flow. Blood is characterized as an incompressible fluid with a density of 1060 kg/m^3^ and a viscosity of 0.003 kg/m-s [17]. The blood fluid characteristic was described as non-Newtonian as set in the software. The inlet boundary conditions were set as a constant and uniform velocity of 0.3 m/s, which is the default setting for this parameter in Ansys Fluent, whereas no precise requirements were given for the outflow. The graft and artery walls were subjected to stiff, no-slip boundary conditions [18]. Subsequently, a hybrid approach was implemented for the initialization procedure. A total of 150 iterations were set for the calculation and analysis. Finally, the optimal diameter, anastomosis angle, and location will be determined by comparing the flow dynamics, wall shear stress (WSS), and turbulence.

## 3. Results

### 3.1. Wall Shear Stress

**Figure 4:**
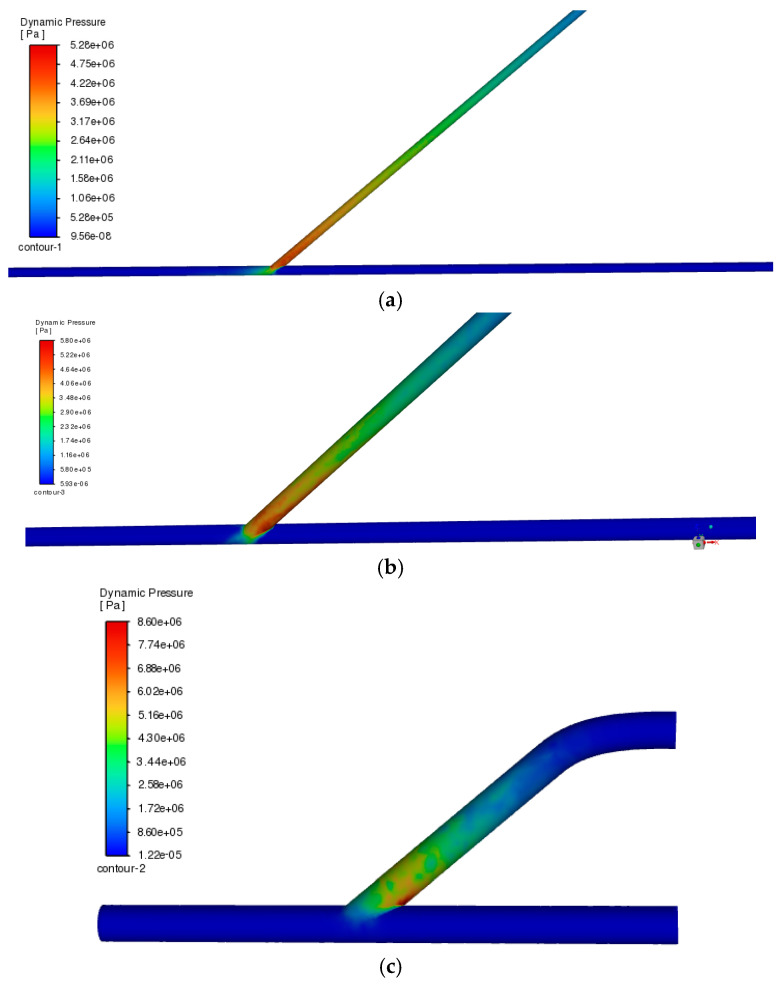
Pressure comparison for the same anastomosis angle with different blood vessel diameters: (**a**) 2 mm, (**b**) 5.1 mm, and (**c**) 14.5 mm. The lesser the red color of the pressure contour along the anastomosis site, the less stress applied and an ideal diameter.

**Figure A1:**
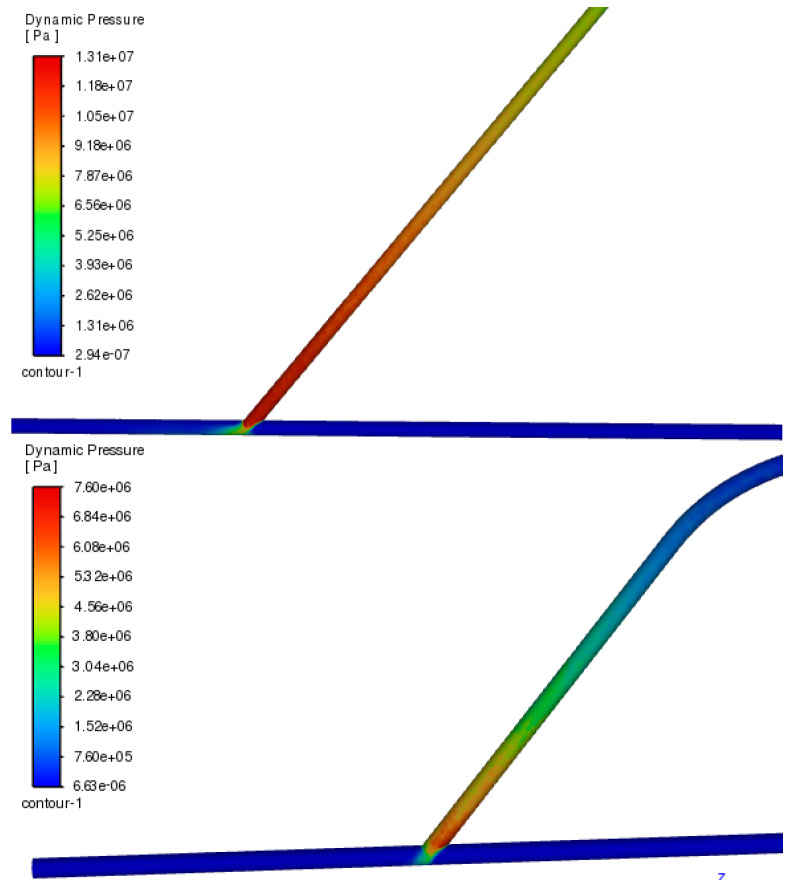

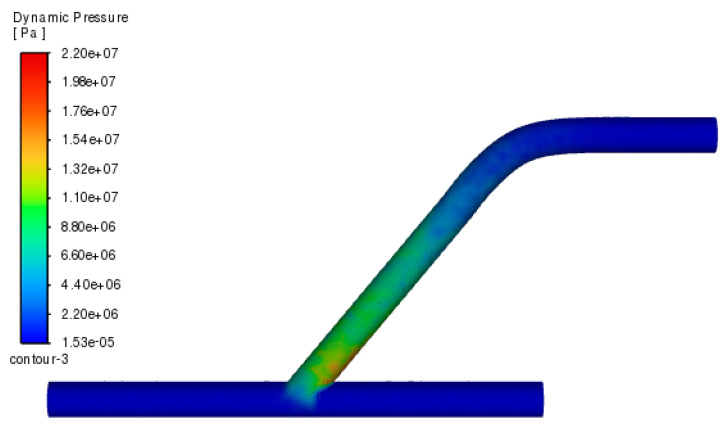
Wall Shear Stress comparison for 50-degree anastomosis angle with different blood vessel diameters.

**Figure A2:**
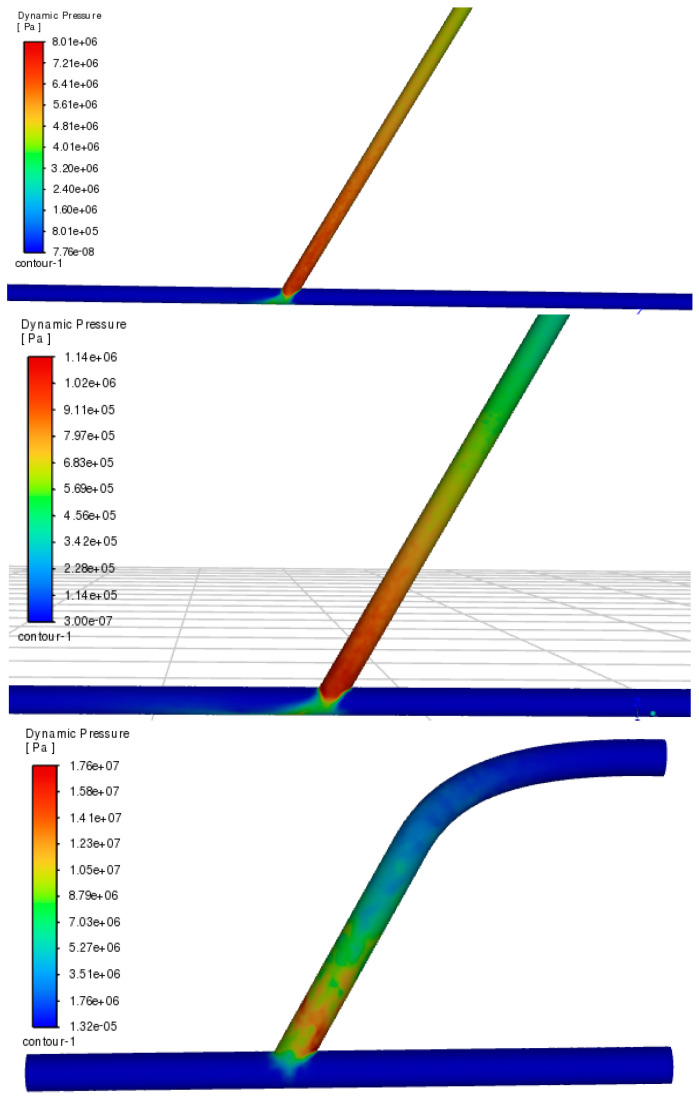
Wall Shear Stress comparison for 60-degree anastomosis angle with different blood vessels.

Figure 4 shows the results for AV grafts having an anastomosis angle of 40° since, comparing the stress contour from its 50° and 60° counterparts, as shown in Figure A1 and Figure A2, respectively, 40° displays the best shearing distribution and less stress applied consistently across all different blood vessel diameters. As such, this optimal angle is suitable for analysis and discussion to fortify which configuration is the best as an access location.

### 3.2. Turbulence

**Figure 5:**
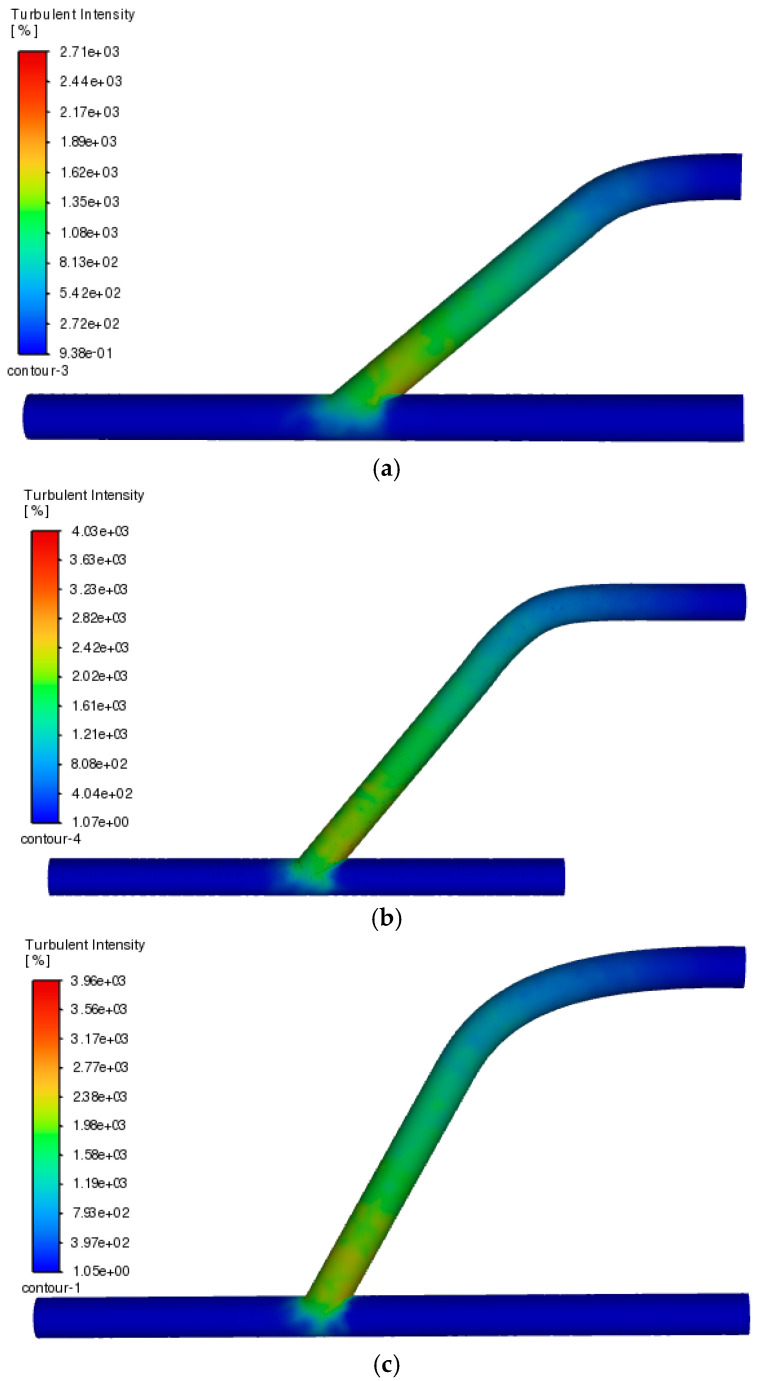
Turbulence comparison for same blood vessel diameters with different anastomosis angles: (**a**) 40°, (**b**) 50°, and (**c**) 60°. The darker the color of red at the conjunction of the attached graft and vein, the better the blood flow.

**Figure A3:**
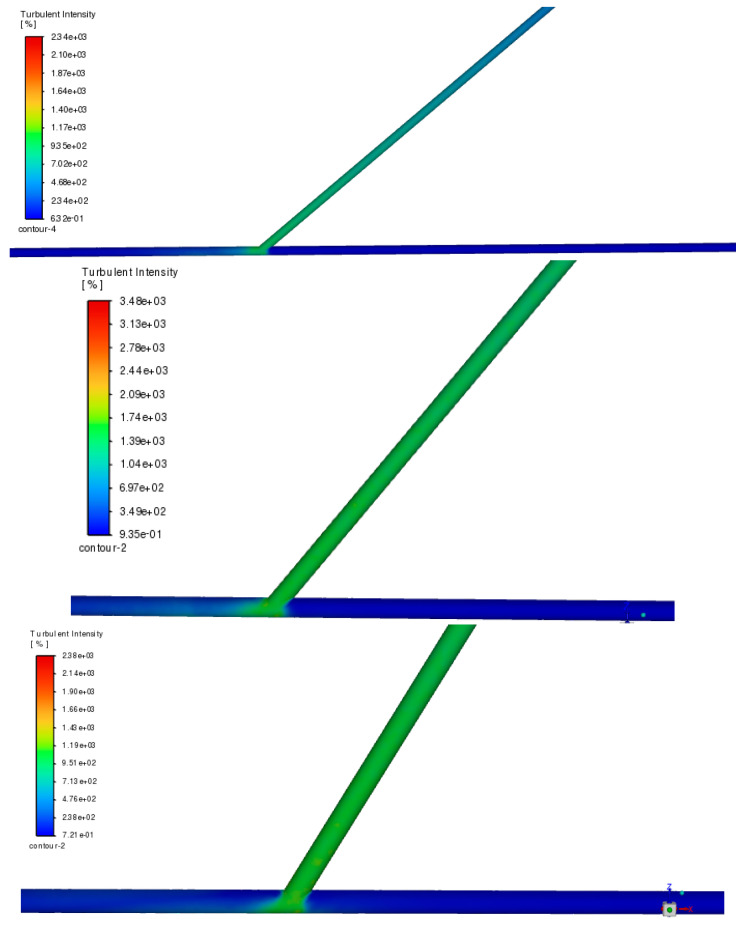
Turbulence comparison for 2 mm wide blood vessels with different anastomosis angles.

**Figure A4:**
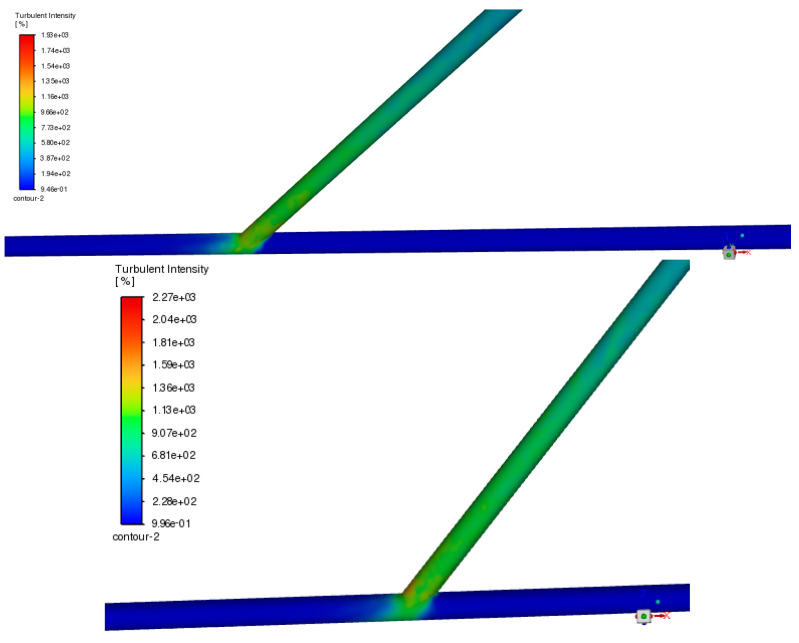

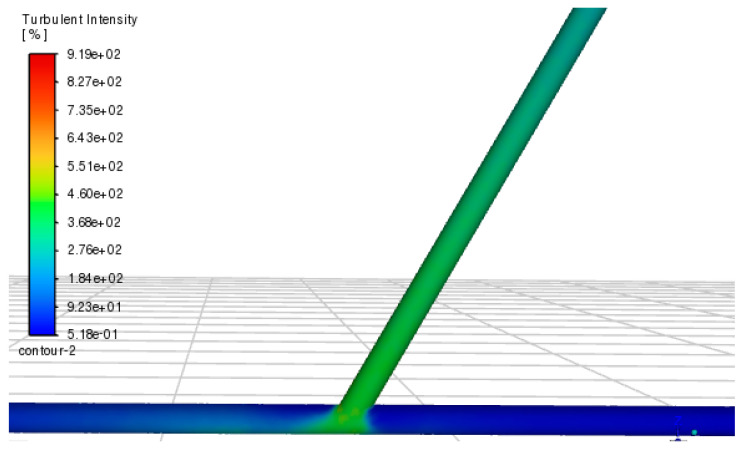
Turbulence comparison for 5.1 mm wide blood vessels with different anastomosis angles.

Similarly, Figure 5 shows the results for AV grafts having a diameter of 14.5 mm, since aside from being the size that was described as the best configuration to allow less stress to occur in the anastomosis site, it may also impose a probable correlation with the turbulence that it may cause as blood passes through the vessel junction. This hypothesis was supported by the results of the Ansys simulation, wherein there is an observable better flow when the diameter of vessels is wider compared to when they are designed to be narrower, as shown in Figure A3 and Figure A4, which correspond to 2 mm and 5.1 mm diameters, respectively.

Having these results may adequately provide an understanding and explanation on how AV grafts function when subjected to different specifications by means of applying CFD, which is a simulated computer-aided design tool that mimics the orientation and configuration of a specific anastomosis site where vascular access will be placed to dialysis patients to optimize which is the best design for the intended purpose.

## 4. Discussion

### 4.1. Wall Shear Stress

One of the most essential parameters to measure in ensuring that blood flow is maintained at a normal pace is to analyze the amount of shearing induced by the movement of blood along the vessel walls. This instance must be monitored since when there is a presence of heavy shearing at a particular spot along wall boundaries, it may produce an accumulation of pressure that will make the wall properties susceptible to unprecedented damage over time, such as rupturing of blood vessels [19]. As such, in deciding which location would be the most suitable area to perform the surgical procedure of creating dialysis access, it must account for the model that would show the least amount of stress induced by the blood flow against the vessel walls to ensure that there would be no or minimal chance of developing endothelial complications caused by the surgical process performed leading to cardiovascular diseases such as thrombosis or embolism.

In identifying wall shear stress, it is important to account for the pressure induced by flowing liquids into the wall boundaries to provide an idea about the density accumulation in every specific area of the blood vessel made in contact with blood [20]. With this, the use of dynamic pressure as a mode of analysis to identify which areas would produce more shearing concerning the junction at which the anastomosis site of the AV graft is attached to the venous portion of the blood vessels at a particular possible access where the implantation may be performed. This fact may be attributed to the contribution of vessel sizes inducing a significant amount of fluid resistance. Thus, it is expected to yield a positive result wherein the lesser the diameter of the vessel, the less the blood flows, which contributes to more resistance, causing a significant amount of shearing, especially within areas where two vessels meet to direct blood flow, as in the case of end-to-side AV graft anastomosis [21]. As such, the role of blood viscosity must be established in the dynamic flow solver. The value used to represent the viscosity of blood is 0.003 kg/m·s, which is within the normal viscosity values being referenced by various cardiovascular handbooks, which are 0.0035 to 0.0055 kg/m·s [22]. In general terms, a higher viscosity value would mean that shearing is lessened since this means that fluid is more liberated to flow along the surface it passes through, thus creating a lesser frictional effect that would promote the retention of fluid at a particular area where it may concentrate if not allowed to flow according to the normal blood flow velocity of about 40 cm/s or 0.4 m/s. However, this study focused on choosing a blood flow that is not in the max velocity to consider variations that may be induced by other parameters (gender and age).

Figure 4 shows the pressure contour along the anastomosis site with varying tube diameter measurements. The model was represented by introducing the blood flow from the inlet portion of the angled graft attached to the horizontal vein orientation and analyzing the effect of the size deviations using identification of the location of massive concentrations of shearing-induced stress in the vessel walls proximal to the angled configuration. The results yielded a unanimous outcome of pressure profile, wherein as the tube diameter is being increased, the less shear stress is applied to the walls by the blood flow. This finding is also consistent with the configurations that were set up as shown in Appendix A—Figure A1 and Figure A2. The relationship between shear stress-induced pressure along vessel walls and the vessel diameter was confirmed by this result, which explains that the better configuration to avoid unprecedented stress accumulation in a specific area along the wall, especially in the anastomosis site, is to choose an area for dialysis access that has the widest diameter among all feasible choices. Another noticeable observation is the effect of the anastomosis angle on the pressure accumulating along the junction site. Following the contour pattern for all configurations with the widest vessel diameter, the 40° anastomosis angle produces the least amount of pressure which corresponds to the walls being subjected to less shear stress. To support and validate this finding, a study conducted by Williams et al. (2021) concerning a similar topic on tailoring the optimal anastomosis angle for graft-to-vein configuration found that at lower angles such as 30° or 40° from the horizontal vein, pressure gradients presented a healthier shear rate compared to angles higher than those values [23]. They also found that there is even a balanced distribution of shearing from the proximal to distal region from the anastomosis site that is within the normal range of pressure exerted by the blood to the walls. They also found that at lower angles than 30°, the pressure induced by the blood flow starts to deviate from the healthy spectra, which would suggest that the optimal angle would be around 30° based on the constraints and conditions that were set.

Having provided all that information, an excellent point of improvement for this study is if angles lower than 40° were to be analyzed as well to test if the results that would be retrieved complement the existing data they presented in their study. Overall, it can be deduced that the optimal angle given the conditions set in the study would be allowing the widest vessel diameter to have an anastomosis junction of 40° to maximize efficient blood flow and avoid excess stress applied to the vein walls.

### 4.2. Turbulence

Turbulence is also another important parameter to be measured when analyzing blood flow across the vessel walls. By theory, it is more favorable to have as little turbulence as possible to allow an efficient blood flow to occur. Having a turbulent nature of flow may put a person at risk of having lesions and coagulation of blood in the area where turbulence is high, which may cause vessels to accumulate along vessel walls, leading to complications such as aneurysm, arteriosclerosis, and stenosis. With this, it is essential to maintain a constant laminar flow of blood, which was found to be the ideal physiological hemodynamic characteristic of the body, to avoid these complications occurring, especially when designing grafts involving the attachment of an artificial vessel to an existing artery or vein [24]. For this section, the intention is to identify the size and angle configuration of the vascular access that produces the least turbulence, particularly along the anastomosis junction and the areas proximal to the inlet vessel going to the outlet vein. This must be considered to ensure that the graft design induces minimal effect and innate perturbation to the nature of fluid flow inside the vessel walls.

Figure 5 shows the turbulence comparison of the graft design model with varying anastomosis angles but with a constant vessel diameter of 14.5 mm by applying the actual fluid flow feature of Ansys Fluent to process and display motion intensity along the simulated vessles. It is noticeable that the level of turbulence in the angled portion of the vessel increases as the angle increases. In addition, the resulting turbulence of the angle going towards the vein junction is more profound in the 60° angle compared to lower angles due to the heavy concentration of turbulence at the exact junction where the graft and vein were attached. This finding is consistent with the study conducted by Ene-Iordache et al. (2012), wherein they found that sharper anastomosis angles have less distribution of flow disturbance area compared to higher angles [25]. This was further acclaimed by their obtaining relevant residence time (RRT) of the fluid in their simulation, wherein lower values would correspond to laminar flow conditions, which is ideal in mimicking the normal blood flow in the body. As such, it would also be better if this study also included calculations of such parameters to validate that turbulence is more intense when anastomosis angles are being increased.

Furthermore, to consider the effect of varying vessel diameter on the flow turbulence, simulations were also done with varying diameters and varying angles as shown in Appendix A—Figure A3 and Figure A4. Following the trend displayed by the figures, it shows that turbulent intensity may have been less profound compared to larger vessel diameters, but the presence of turbulence remains evident even at areas further going to the outlet vein portion. Thus, this would signify that there is inconsistency in blood flow even at areas outside the bounds of the anastomosis site, which is not an ideal model to visualize the intended graft design. This finding is also complimented by a proposition stated in the physiology of circulation that the lesser the area for the fluids to flow, the more chances they may have contact with the wall barriers, thus promoting turbulence as they bounce back and forth following the flow path [26]. With all these things gathered, it is deduced that the best candidate for the graft design is the model that shows the largest vessel diameter and the lowest anastomosis angle of attachment.

Table 1 summarizes the result of the fluid dynamic analysis performed to fulfill the study’s objectives. In this regard, it can be materialized that the best design for the arterial vascular graft should have the characteristic of having a diameter of 14.5 mm, inclined to an angle of 40° from the vein vessel following an end-to-side anastomosis configuration. This idea also explains that the best site for vascular access in dialysis treatment is to implant the graft on the brachiocephalic area or the forearm part of the arm. Based on hemodialysis related literature, this area is also the common location where grafts are implanted due to its potency of giving an opportunity for future graft placement to be performed in case of failure of subsequent graft replacements in the forearm [27].

## 5. Conclusions

For individuals with end-stage renal failure, which requires dialysis to maintain their body free from harmful wastes to be recirculated along the bloodstream, a functional AV graft is crucial for ensuring efficient blood flow during treatment. This study employed CFD analysis to identify the optimal design parameters for AV grafts, minimizing complications and promoting efficient blood flow. The analysis focused on two key factors: wall shear stress and turbulence. Wall shear stress is the frictional force exerted by blood flow on the vessel walls. Excessive shear stress can damage the vessel walls, potentially leading to complications like aneurysms. Turbulence, on the other hand, refers to irregular blood flow patterns. High turbulence can promote blood clotting and vessel narrowing.

This study investigated the impact of two design variables: vessel diameter and anastomosis angle. The anastomosis angle refers to the angle at which the AV graft connects to the vein. The findings revealed that larger vessel diameters (14.5 mm) experienced less wall shear stress compared to smaller diameters. Additionally, lower anastomosis angles (40°) resulted in less pressure on the vessel walls and lower turbulence levels within the graft, compared to higher angles. These findings suggest that the ideal AV graft design for dialysis access should have a diameter of 14.5 mm and an anastomosis angle of 40°. This configuration minimizes both wall shear stress and turbulence, promoting efficient blood flow and potentially reducing the risk of complications associated with dialysis access, such as blood vessel damage and clotting. This study also identified potential areas for further investigation. Overall, by refining AV graft design based on these findings and future research, we can improve outcomes for dialysis patients by minimizing complications and ensuring efficient blood flow during treatment.

Future research could incorporate calculations of RRT to further validate the turbulence findings. Varying blood flow velocity could be explored for more robust design recommendations. Anastomosis angles lower than 40° could provide additional insights into their impact on blood flow dynamics. Iterations of this study could explore other computational models specified for patients whose dominant arm is the left one, which necessitates the use of the right arm for implant procedures and hemodynamic conditions for individualized treatments. The effect of different graft diameters and angles on thrombosis, arteriosclerosis, and other relevant complications can also be explored to evaluate unprecedented risks for subsequent guidance through future vascular remodeling. Lastly, future research could conduct comparative studies with alternative vascular access methods like AV fistulas to evaluate efficacy.

## Figures and Tables

**Table 1:** Summary of the CFD results for Various AV Graft Diameter and Configuration (Wall Shear Stress and Turbulence Assessment).

	A: 40°, D: 2 mm	A: 40°, D: 5.1 mm	A: 40°, D: 14.5 mm	A: 50°, D: 2 mm	A: 50°, D: 5.1 mm	A: 50°, D: 14.5 mm	A: 60°, D: 2 mm	A: 60°, D: 5.1 mm	A: 60°, D: 14.5 mm
Wall Shear Stress	A	A	IDEAL	X	X	D	X	X	D
Turbulence	A	A	IDEAL	X	X	D	X	X	D

Legend: A/D: Only passed the specified ideal graft design parameters; candidate alternative; X: not suitable for ideal graft design.

## Data Availability

The raw data supporting the conclusions of this article will be made available by the authors on request.
